# Supplementary material for: Iterative improvement in the automatic modular design of robot swarms
Source: PeerJ Comput Sci. 2020 Dec 7;6:e322. doi: 10.7717/peerj-cs.322 (PMC7924708; doi:10.7717/peerj-cs.322)
Supplement: Supplemental Information 3 [file peerj-cs-06-322-s003.zip › argos3/doc/api/standalone/a00660_source.html]

ARGoS: plugins/simulator/visualizations/qt-opengl/qtopengl\_widget.cpp Source File


- Main Page
- Related Pages
- Namespaces
- Classes
- Files

- File List
- File Members

# plugins/simulator/visualizations/qt-opengl/qtopengl\_widget.cpp

Go to the documentation of this file.

```
00001 
00007 #include "qtopengl_widget.h"
00008 #include "qtopengl_main_window.h"
00009 #include "qtopengl_user_functions.h"
00010 
00011 #include <argos3/core/utility/logging/argos_log.h>
00012 #include <argos3/core/utility/math/plane.h>
00013 #include <argos3/core/simulator/simulator.h>
00014 #include <argos3/core/simulator/loop_functions.h>
00015 #include <argos3/core/simulator/space/space.h>
00016 #include <argos3/core/simulator/entity/floor_entity.h>
00017 #include <argos3/core/simulator/entity/composable_entity.h>
00018 #include <argos3/core/simulator/entity/positional_entity.h>
00019 
00020 #include <QDir>
00021 #include <QToolTip>
00022 #include <QTimerEvent>
00023 #include <QMouseEvent>
00024 #include <QPainter>
00025 
00026 #ifndef GL_MULTISAMPLE
00027 #define GL_MULTISAMPLE 0x809D
00028 #endif
00029 
00030 namespace argos {
00031 
00032    static const Real ASPECT_RATIO         = 4.0f / 3.0f;
00033    static const UInt32 SELECT_BUFFER_SIZE = 128;
00034    
00035    /****************************************/
00036    /****************************************/
00037 
00038    CQTOpenGLWidget::CQTOpenGLWidget(QWidget* pc_parent,
00039                                     CQTOpenGLMainWindow& c_main_window,
00040                                     CQTOpenGLUserFunctions& c_user_functions) :
00041       QOpenGLWidget(pc_parent),
00042       m_cMainWindow(c_main_window),
00043       m_cUserFunctions(c_user_functions),
00044       nTimerId(-1),
00045       m_bFastForwarding(false),
00046       m_nDrawFrameEvery(1),
00047       m_nFrameCounter(0),
00048       m_bMouseGrabbed(false),
00049       m_bShiftPressed(false),
00050       m_bInvertMouse(false),
00051       m_cSimulator(CSimulator::GetInstance()),
00052       m_cSpace(m_cSimulator.GetSpace()),
00053       m_bUsingFloorTexture(false),
00054       m_pcFloorTexture(NULL),
00055       m_pcGroundTexture(NULL),
00056       m_punSelectionBuffer(new GLuint[SELECT_BUFFER_SIZE])
00057    {
00058       /* Set the widget's size policy */
00059       QSizePolicy cSizePolicy(QSizePolicy::Preferred, QSizePolicy::Preferred);
00060       cSizePolicy.setHeightForWidth(true);
00061       setSizePolicy(cSizePolicy);
00062       /* Grab focus when clicked on */
00063       setFocusPolicy(Qt::ClickFocus);
00064       /* Force size and geometry */
00065       updateGeometry();
00066       /* Keys */
00067       m_mapPressedKeys[DIRECTION_UP]        = false;
00068       m_mapPressedKeys[DIRECTION_DOWN]      = false;
00069       m_mapPressedKeys[DIRECTION_LEFT]      = false;
00070       m_mapPressedKeys[DIRECTION_RIGHT]     = false;
00071       m_mapPressedKeys[DIRECTION_FORWARDS]  = false;
00072       m_mapPressedKeys[DIRECTION_BACKWARDS] = false;
00073    }
00074 
00075    /****************************************/
00076    /****************************************/
00077 
00078    CQTOpenGLWidget::~CQTOpenGLWidget() {
00079       makeCurrent();
00080       delete m_pcGroundTexture;
00081       glDeleteLists(1, m_unArenaList);
00082       if(m_bUsingFloorTexture) {
00083          delete m_pcFloorTexture;
00084          glDeleteLists(1, m_unFloorList);
00085       }
00086       delete[] m_punSelectionBuffer;
00087       doneCurrent();
00088    }
00089 
00090    /****************************************/
00091    /****************************************/
00092 
00093    void CQTOpenGLWidget::initializeGL() {
00094       /* Initializes the openGL functions */
00095       initializeOpenGLFunctions();
00096       /* Set clear color */
00097       glClearColor(0, .5, .5, 255); // dark cyan
00098       /* Set up the texture parameters for the floor plane
00099          (here we refer to the standard floor, not the floor entity) */
00100       m_pcGroundTexture = new QOpenGLTexture(QImage(m_cMainWindow.GetTextureDir() + "/ground.png"));
00101       m_pcGroundTexture->setMinMagFilters(QOpenGLTexture::LinearMipMapLinear,
00102                                           QOpenGLTexture::Linear);
00103 #ifdef ARGOS_WITH_FREEIMAGE
00104       /* Now take care of the floor entity */
00105       try {
00106          /* Create an image to use as texture */
00107          m_cSpace.GetFloorEntity().SaveAsImage("/tmp/argos_floor.png");
00108          m_bUsingFloorTexture = true;
00109          /* Use the image as texture */
00110          m_pcFloorTexture = new QOpenGLTexture(QImage("/tmp/argos_floor.png"));
00111          m_pcFloorTexture->setMinMagFilters(QOpenGLTexture::LinearMipMapLinear,
00112                                             QOpenGLTexture::Linear);
00113          m_cSpace.GetFloorEntity().ClearChanged();
00114       }
00115       catch(CARGoSException& ex) {}
00116 #endif
00117       /* Nicest hints */
00118       glHint(GL_PERSPECTIVE_CORRECTION_HINT, GL_NICEST);
00119       glHint(GL_TEXTURE_COMPRESSION_HINT, GL_NICEST);
00120       /* Setup lighting */
00121       GLfloat pfLightAmbient[]  = {   .2,   .2,  .2, 1. };
00122       GLfloat pfLightDiffuse[]  = {   .8,   .8,  .8, 1. };
00123       GLfloat pfLightPosition[] = { 50. , 50. , 2. , 1. };
00124       glLightfv(GL_LIGHT0, GL_AMBIENT,  pfLightAmbient);
00125       glLightfv(GL_LIGHT0, GL_DIFFUSE,  pfLightDiffuse);
00126       glLightfv(GL_LIGHT0, GL_POSITION, pfLightPosition);
00127       glEnable(GL_LIGHT0);
00128    }
00129 
00130    /****************************************/
00131    /****************************************/
00132 
00133    void CQTOpenGLWidget::paintGL() {
00134       /* Clear accumulator buffer */
00135       glClearAccum(0.0, 0.0, 0.0, 0.0);
00136       /* Clear color buffer and depth buffer */
00137       glClear(GL_COLOR_BUFFER_BIT | GL_DEPTH_BUFFER_BIT);
00138       /* Smooth lines and shades */
00139       glEnable(GL_LINE_SMOOTH);
00140       glShadeModel(GL_SMOOTH);
00141       /* Enable depth testing */
00142       glEnable(GL_DEPTH_TEST);
00143       /* Enable face culling */
00144       glEnable(GL_CULL_FACE);
00145       /* Enable lighting */
00146       glEnable(GL_LIGHTING);
00147       /* Calculate the perspective matrix */
00148       glMatrixMode(GL_PROJECTION);
00149       glLoadIdentity();
00150       gluPerspective(m_cCamera.GetActiveSettings().YFieldOfView.GetValue(),
00151                      ASPECT_RATIO,
00152                      0.1f, 1000.0f);
00153       /* Place the camera */
00154       glMatrixMode(GL_MODELVIEW);
00155       glLoadIdentity();
00156       m_cCamera.Look();
00157       /* Draw the arena */
00158       DrawArena();
00159       /* Draw the objects */
00160       CEntity::TVector& vecEntities = m_cSpace.GetRootEntityVector();
00161       for(CEntity::TVector::iterator itEntities = vecEntities.begin();
00162           itEntities != vecEntities.end();
00163           ++itEntities) {
00164          glPushMatrix();
00165          CallEntityOperation<CQTOpenGLOperationDrawNormal, CQTOpenGLWidget, void>(*this, **itEntities);
00166          m_cUserFunctions.Call(**itEntities);
00167          glPopMatrix();
00168       }
00169       /* Draw the selected object, if necessary */
00170       if(m_sSelectionInfo.IsSelected) {
00171          glPushMatrix();
00172          CallEntityOperation<CQTOpenGLOperationDrawSelected, CQTOpenGLWidget, void>(*this, *vecEntities[m_sSelectionInfo.Index]);
00173          glPopMatrix();
00174       }
00175       /* Draw in world */
00176       glPushMatrix();
00177       m_cUserFunctions.DrawInWorld();
00178       glPopMatrix();
00179       /* Draw axes */
00180       DrawAxes();
00181       /* Draw selection ray for debug */
00182       glDisable(GL_LIGHTING);
00183       glLineWidth(1.0f);
00184       glBegin(GL_LINES);
00185       glColor3f(1.0, 0.0, 0.0);
00186       const CVector3& cStart = m_cSelectionRay.GetStart();
00187       const CVector3& cEnd = m_cSelectionRay.GetEnd();
00188       glVertex3f(cStart.GetX(), cStart.GetY(), cStart.GetZ());
00189       glVertex3f(cEnd.GetX(), cEnd.GetY(), cEnd.GetZ());
00190       glEnd();
00191       glEnable(GL_LIGHTING);
00192       /* Execute overlay drawing */
00193       glShadeModel(GL_FLAT);
00194       glDisable(GL_LIGHTING);
00195       glDisable(GL_CULL_FACE);
00196       glDisable(GL_DEPTH_TEST);
00197       glMatrixMode(GL_MODELVIEW);
00198       QPainter cPainter(this);
00199       cPainter.setRenderHint(QPainter::Antialiasing);
00200       cPainter.setRenderHint(QPainter::TextAntialiasing);
00201       m_cUserFunctions.DrawOverlay(cPainter);
00202       // cPainter.drawText(rect(), QString("%1 FPS").arg(m_fFPS, 0, 'f', 0));
00203       cPainter.end();
00204       /* Grab frame, if necessary */
00205       if(m_sFrameGrabData.Grabbing) {
00206          QString strFileName = QString("%1/%2%3.%4")
00207             .arg(m_sFrameGrabData.Directory)
00208             .arg(m_sFrameGrabData.BaseName)
00209             .arg(m_cSpace.GetSimulationClock(), 5, 10, QChar('0'))
00210             .arg(m_sFrameGrabData.Format);
00211          QToolTip::showText(pos() + geometry().center(), "Stored frame to \"" + strFileName);
00212          grabFramebuffer()
00213             .save(
00214                strFileName,
00215                0,
00216                m_sFrameGrabData.Quality);
00217       }
00218    }
00219 
00220    /****************************************/
00221    /****************************************/
00222 
00223    CRay3 CQTOpenGLWidget::RayFromWindowCoord(int n_x,
00224                                              int n_y) {
00225       /* Make sure OpenGL context is correct */
00226       makeCurrent();
00227       /* Rescale coordinates by devicePixelRatio */
00228       n_x *= devicePixelRatio();
00229       n_y *= devicePixelRatio();
00230       /* Get current viewport */
00231       GLint nViewport[4];
00232       glGetIntegerv(GL_VIEWPORT, nViewport);
00233       /* Get OpenGL matrices */
00234       GLdouble fModelViewMatrix[16];
00235       GLdouble fProjectionMatrix[16];
00236       glGetDoublev(GL_MODELVIEW_MATRIX, fModelViewMatrix);
00237       glGetDoublev(GL_PROJECTION_MATRIX, fProjectionMatrix);
00238       /*
00239        * Convert mouse position in window into OpenGL representation
00240        */
00241       /* The x coordinate stays the same */
00242       GLfloat fWinX = n_x;
00243       /* The y coordinate of the window is top-left; in OpenGL is bottom-left */
00244       GLfloat fWinY = nViewport[3] - n_y;
00245       /*
00246        * Get the position of the ray start in the world
00247        * The ray starts at the near clipping plane (depth = 0.0f)
00248        */
00249       GLdouble fRayStartX, fRayStartY, fRayStartZ;
00250       gluUnProject(fWinX, fWinY, 0.0f,
00251                    fModelViewMatrix, fProjectionMatrix, nViewport,
00252                    &fRayStartX, &fRayStartY, &fRayStartZ);
00253       /*
00254        * Get the position of the ray end in the world
00255        * The ray starts at the far clipping plane (depth = 1.0f)
00256        */
00257       GLdouble fRayEndX, fRayEndY, fRayEndZ;
00258       gluUnProject(fWinX, fWinY, 1.0f,
00259                    fModelViewMatrix, fProjectionMatrix, nViewport,
00260                    &fRayEndX, &fRayEndY, &fRayEndZ);
00261       doneCurrent();
00262       return CRay3(CVector3(fRayStartX, fRayStartY, fRayStartZ),
00263                    CVector3(fRayEndX, fRayEndY, fRayEndZ));
00264    }
00265 
00266    /****************************************/
00267    /****************************************/
00268 
00269    CVector3 CQTOpenGLWidget::GetWindowCoordInWorld(int n_x,
00270                                                    int n_y) {
00271       /* Make sure OpenGL context is correct */
00272       makeCurrent();
00273       /* Rescale coordinates by devicePixelRatio */
00274       n_x *= devicePixelRatio();
00275       n_y *= devicePixelRatio();
00276       /* Get current viewport */
00277       GLint nViewport[4];
00278       glGetIntegerv(GL_VIEWPORT, nViewport);
00279       /* Get OpenGL matrices */
00280       GLdouble fModelViewMatrix[16];
00281       GLdouble fProjectionMatrix[16];
00282       glGetDoublev(GL_MODELVIEW_MATRIX, fModelViewMatrix);
00283       glGetDoublev(GL_PROJECTION_MATRIX, fProjectionMatrix);
00284       /*
00285        * Convert mouse position in window into a 3D representation
00286        */
00287       /* The x coordinate stays the same */
00288       GLfloat fWinX = n_x;
00289       /* The y coordinate of the window is top-left; in OpenGL is bottom-left */
00290       GLfloat fWinY = nViewport[3] - n_y;
00291       /* Read the z coordinate from the depth buffer in the back buffer */
00292       GLfloat fWinZ;
00293       glReadBuffer(GL_BACK);
00294       glReadPixels(n_x, (GLint)fWinY, 1, 1, GL_DEPTH_COMPONENT, GL_FLOAT, &fWinZ);
00295       /* Get the actual position in the world */
00296       GLdouble fWorldX, fWorldY, fWorldZ;
00297       gluUnProject(fWinX, fWinY, fWinZ,
00298                    fModelViewMatrix, fProjectionMatrix, nViewport,
00299                    &fWorldX, &fWorldY, &fWorldZ);
00300       /*
00301        * Swap coordinates when creating the ray
00302        * In ARGoS, the up vector is the Z-axis, while in OpenGL it is the Y-axis
00303        */
00304       doneCurrent();
00305       return CVector3(fWorldX, fWorldZ, fWorldY);
00306    }
00307 
00308    /****************************************/
00309    /****************************************/
00310 
00311    CEntity* CQTOpenGLWidget::GetSelectedEntity() {
00312       return (m_sSelectionInfo.IsSelected ?
00313               m_cSpace.GetRootEntityVector()[m_sSelectionInfo.Index] :
00314               NULL);
00315    }
00316 
00317    /****************************************/
00318    /****************************************/
00319 
00320    void CQTOpenGLWidget::SelectEntity(CEntity& c_entity) {
00321       /* Look for the idx corresponding to the entity */
00322       size_t unIdx = 0;
00323       while(m_cSpace.GetRootEntityVector()[unIdx] != &c_entity)
00324          ++unIdx;
00325       /* Check whether an entity had previously been selected */
00326       if(m_sSelectionInfo.IsSelected) {
00327          /* An entity had previously been selected */
00328          /* Is that entity already selected? */
00329          if(m_sSelectionInfo.Index == unIdx) return;
00330          /* Deselect the previous one */
00331          emit EntityDeselected(m_sSelectionInfo.Index);
00332          m_cUserFunctions.EntityDeselected(
00333             *m_cSpace.GetRootEntityVector()[m_sSelectionInfo.Index]);
00334       }
00335       else {
00336          /* No entity had previously been selected */
00337          m_sSelectionInfo.IsSelected = true;
00338       }
00339       /* Select the new entity */
00340       m_sSelectionInfo.Index = unIdx;
00341       emit EntitySelected(unIdx);
00342       m_cUserFunctions.EntitySelected(
00343          *m_cSpace.GetRootEntityVector()[unIdx]);
00344       update();
00345    }
00346 
00347    /****************************************/
00348    /****************************************/
00349 
00350    void CQTOpenGLWidget::DeselectEntity() {
00351       /* If no entity was selected, nothing to do */
00352       if(!m_sSelectionInfo.IsSelected) return;
00353       /* Deselect the entity */
00354       emit EntityDeselected(m_sSelectionInfo.Index);
00355       m_cUserFunctions.EntityDeselected(
00356          *m_cSpace.GetRootEntityVector()[m_sSelectionInfo.Index]);
00357       m_sSelectionInfo.IsSelected = false;
00358       update();
00359    }
00360 
00361    /****************************************/
00362    /****************************************/
00363 
00364    void CQTOpenGLWidget::SelectInScene(UInt32 un_x,
00365                                        UInt32 un_y) {
00366       /* Make sure OpenGL context is correct */
00367       makeCurrent();
00368       un_x *= devicePixelRatio();
00369       un_y *= devicePixelRatio();
00370       /* Used to store the viewport size */
00371       GLint nViewport[4];
00372       /* Set the selection buffer */
00373       glSelectBuffer(SELECT_BUFFER_SIZE, m_punSelectionBuffer);
00374       /* Switch to select mode */
00375       glRenderMode(GL_SELECT);
00376       /* Set the projection matrix */
00377       glMatrixMode(GL_PROJECTION);
00378       glLoadIdentity();
00379       /* Set the viewport */
00380       glGetIntegerv(GL_VIEWPORT, nViewport);
00381       gluPickMatrix(un_x,
00382                     nViewport[3]-un_y,
00383                     5, 5,
00384                     nViewport);
00385       gluPerspective(m_cCamera.GetActiveSettings().YFieldOfView.GetValue(),
00386                      ASPECT_RATIO,
00387                      0.1f, 1000.0f);
00388       glMatrixMode(GL_MODELVIEW);
00389       glLoadIdentity();
00390       m_cCamera.Look();
00391       /* Prepare name stack */
00392       glInitNames();
00393       /* Draw the objects */
00394       CEntity::TVector& vecEntities = m_cSpace.GetRootEntityVector();
00395       for(size_t i = 0; i < vecEntities.size(); ++i) {
00396          glPushName(i);
00397          glPushMatrix();
00398          CallEntityOperation<CQTOpenGLOperationDrawNormal, CQTOpenGLWidget, void>(*this, *vecEntities[i]);
00399          glPopMatrix();
00400          glPopName();
00401       }
00402       glFlush();
00403       /* Return to normal rendering mode and get hit count */
00404       bool bWasSelected = m_sSelectionInfo.IsSelected;
00405       UInt32 unHits = glRenderMode(GL_RENDER);
00406       if (unHits == 0) {
00407          /* No hits, deselect what was selected */
00408          m_sSelectionInfo.IsSelected = false;
00409          if(bWasSelected) {
00410             emit EntityDeselected(m_sSelectionInfo.Index);
00411             m_cUserFunctions.EntityDeselected(
00412                *m_cSpace.GetRootEntityVector()[m_sSelectionInfo.Index]);
00413          }
00414       }
00415       else {
00416          /* There are hits!
00417           * Process them, keeping the closest hit
00418           */
00419          GLuint* punByte = m_punSelectionBuffer;
00420          GLuint unMinZ = 0xffffffff;
00421          GLuint* punName = NULL;
00422          for (UInt32 i = 0; i < unHits; i++) {  
00423             GLuint unNames = *punByte;
00424             ++punByte;
00425             if (*punByte < unMinZ) {
00426                unMinZ = *punByte;
00427                punName = punByte+2;
00428             }
00429             punByte += unNames+2;
00430          }
00431          /* Now *punName contains the closest hit */
00432          if(bWasSelected &&
00433             (m_sSelectionInfo.Index == *punName)) {
00434             /* The user clicked on the selected entity, deselect it */
00435             emit EntityDeselected(m_sSelectionInfo.Index);
00436             m_sSelectionInfo.IsSelected = false;
00437             m_cUserFunctions.EntitySelected(
00438                *m_cSpace.GetRootEntityVector()[m_sSelectionInfo.Index]);
00439          }
00440          if(bWasSelected &&
00441             (m_sSelectionInfo.Index != *punName)) {
00442             /* The user clicked on a different entity from the selected one */
00443             emit EntityDeselected(m_sSelectionInfo.Index);
00444             m_cUserFunctions.EntityDeselected(
00445                *m_cSpace.GetRootEntityVector()[m_sSelectionInfo.Index]);
00446             m_sSelectionInfo.Index = *punName;
00447             emit EntitySelected(m_sSelectionInfo.Index);
00448             m_cUserFunctions.EntitySelected(
00449                *m_cSpace.GetRootEntityVector()[m_sSelectionInfo.Index]);
00450          }
00451          else {
00452             /* There was nothing selected, and the user clicked on an entity */
00453             m_sSelectionInfo.IsSelected = true;
00454             m_sSelectionInfo.Index = *punName;
00455             emit EntitySelected(m_sSelectionInfo.Index);
00456             m_cUserFunctions.EntitySelected(
00457                *m_cSpace.GetRootEntityVector()[m_sSelectionInfo.Index]);
00458          }
00459       }
00460       doneCurrent();
00461       /* Redraw */
00462       update();
00463    }
00464 
00465    /****************************************/
00466    /****************************************/
00467 
00468    void CQTOpenGLWidget::DrawEntity(CPositionalEntity& c_entity) {
00469       /* Get the position of the entity */
00470       const CVector3& cPosition = c_entity.GetPosition();
00471       /* Get the orientation of the entity */
00472       const CQuaternion& cOrientation = c_entity.GetOrientation();
00473       CRadians cZAngle, cYAngle, cXAngle;
00474       cOrientation.ToEulerAngles(cZAngle, cYAngle, cXAngle);
00475       /* First, translate the entity */
00476       glTranslatef(cPosition.GetX(), cPosition.GetY(), cPosition.GetZ());
00477       /* Second, rotate the entity */
00478       glRotatef(ToDegrees(cXAngle).GetValue(), 1.0f, 0.0f, 0.0f);
00479       glRotatef(ToDegrees(cYAngle).GetValue(), 0.0f, 1.0f, 0.0f);
00480       glRotatef(ToDegrees(cZAngle).GetValue(), 0.0f, 0.0f, 1.0f);
00481    }
00482 
00483    /****************************************/
00484    /****************************************/
00485 
00486    void CQTOpenGLWidget::DrawEntity(CEmbodiedEntity& c_entity) {
00487       /* Get the position of the entity */
00488       const CVector3& cPosition = c_entity.GetOriginAnchor().Position;
00489       /* Get the orientation of the entity */
00490       const CQuaternion& cOrientation = c_entity.GetOriginAnchor().Orientation;
00491       CRadians cZAngle, cYAngle, cXAngle;
00492       cOrientation.ToEulerAngles(cZAngle, cYAngle, cXAngle);
00493       /* First, translate the entity */
00494       glTranslatef(cPosition.GetX(), cPosition.GetY(), cPosition.GetZ());
00495       /* Second, rotate the entity */
00496       glRotatef(ToDegrees(cXAngle).GetValue(), 1.0f, 0.0f, 0.0f);
00497       glRotatef(ToDegrees(cYAngle).GetValue(), 0.0f, 1.0f, 0.0f);
00498       glRotatef(ToDegrees(cZAngle).GetValue(), 0.0f, 0.0f, 1.0f);
00499    }
00500 
00501    /****************************************/
00502    /****************************************/
00503 
00504    void CQTOpenGLWidget::DrawRays(CControllableEntity& c_entity) {
00505       if(! c_entity.GetCheckedRays().empty()) {
00506          glDisable(GL_LIGHTING);
00507          glLineWidth(1.0f);
00508          glBegin(GL_LINES);
00509          for(UInt32 i = 0; i < c_entity.GetCheckedRays().size(); ++i) {
00510             if(c_entity.GetCheckedRays()[i].first) {
00511                glColor3f(1.0, 0.0, 1.0);
00512             }
00513             else {
00514                glColor3f(0.0, 1.0, 1.0);
00515             }
00516             const CVector3& cStart = c_entity.GetCheckedRays()[i].second.GetStart();
00517             const CVector3& cEnd = c_entity.GetCheckedRays()[i].second.GetEnd();
00518             glVertex3f(cStart.GetX(), cStart.GetY(), cStart.GetZ());
00519             glVertex3f(cEnd.GetX(), cEnd.GetY(), cEnd.GetZ());
00520          }
00521          glEnd();
00522          glPointSize(5.0);
00523          glColor3f(0.0, 0.0, 0.0);
00524          glBegin(GL_POINTS);
00525          for(UInt32 i = 0; i < c_entity.GetIntersectionPoints().size(); ++i) {
00526             const CVector3& cPoint = c_entity.GetIntersectionPoints()[i];
00527             glVertex3f(cPoint.GetX(), cPoint.GetY(), cPoint.GetZ());
00528          }
00529          glEnd();
00530          glPointSize(1.0);
00531          glEnable(GL_LIGHTING);
00532       }
00533    }
00534 
00535    /****************************************/
00536    /****************************************/
00537 
00538    void CQTOpenGLWidget::DrawBoundingBox(CEmbodiedEntity& c_entity) {
00539       const SBoundingBox& sBBox = c_entity.GetBoundingBox();
00540       glPolygonMode(GL_FRONT_AND_BACK, GL_LINE);
00541       glDisable(GL_LIGHTING);
00542       glLineWidth(3.0f);
00543       glColor3f(1.0f, 1.0f, 1.0f);
00544       /* This part covers the top and bottom faces (parallel to XY) */
00545       glBegin(GL_QUADS);
00546       /* Bottom face */
00547       glNormal3f(0.0f, 0.0f, -1.0f);
00548       glVertex3f(sBBox.MinCorner.GetX(), sBBox.MinCorner.GetY(), sBBox.MinCorner.GetZ());
00549       glVertex3f(sBBox.MinCorner.GetX(), sBBox.MaxCorner.GetY(), sBBox.MinCorner.GetZ());
00550       glVertex3f(sBBox.MaxCorner.GetX(), sBBox.MaxCorner.GetY(), sBBox.MinCorner.GetZ());
00551       glVertex3f(sBBox.MaxCorner.GetX(), sBBox.MinCorner.GetY(), sBBox.MinCorner.GetZ());
00552       /* Top face */
00553       glNormal3f(0.0f, 0.0f, 1.0f);
00554       glVertex3f(sBBox.MinCorner.GetX(), sBBox.MinCorner.GetY(), sBBox.MaxCorner.GetZ());
00555       glVertex3f(sBBox.MaxCorner.GetX(), sBBox.MinCorner.GetY(), sBBox.MaxCorner.GetZ());
00556       glVertex3f(sBBox.MaxCorner.GetX(), sBBox.MaxCorner.GetY(), sBBox.MaxCorner.GetZ());
00557       glVertex3f(sBBox.MinCorner.GetX(), sBBox.MaxCorner.GetY(), sBBox.MaxCorner.GetZ());
00558       glEnd();
00559       /* This part covers the faces (South, East, North, West) */
00560       glBegin(GL_QUADS);
00561       /* South face */
00562       glNormal3f(-1.0f, 0.0f, 0.0f);
00563       glVertex3f(sBBox.MinCorner.GetX(), sBBox.MinCorner.GetY(), sBBox.MinCorner.GetZ());
00564       glVertex3f(sBBox.MinCorner.GetX(), sBBox.MinCorner.GetY(), sBBox.MaxCorner.GetZ());
00565       glVertex3f(sBBox.MinCorner.GetX(), sBBox.MaxCorner.GetY(), sBBox.MaxCorner.GetZ());
00566       glVertex3f(sBBox.MinCorner.GetX(), sBBox.MaxCorner.GetY(), sBBox.MinCorner.GetZ());
00567       /* East face */
00568       glNormal3f(0.0f, -1.0f, 0.0f);
00569       glVertex3f(sBBox.MinCorner.GetX(), sBBox.MinCorner.GetY(), sBBox.MinCorner.GetZ());
00570       glVertex3f(sBBox.MaxCorner.GetX(), sBBox.MinCorner.GetY(), sBBox.MinCorner.GetZ());
00571       glVertex3f(sBBox.MaxCorner.GetX(), sBBox.MinCorner.GetY(), sBBox.MaxCorner.GetZ());
00572       glVertex3f(sBBox.MinCorner.GetX(), sBBox.MinCorner.GetY(), sBBox.MaxCorner.GetZ());
00573       /* North face */
00574       glNormal3f(1.0f, 0.0f, 0.0f);
00575       glVertex3f(sBBox.MaxCorner.GetX(), sBBox.MinCorner.GetY(), sBBox.MinCorner.GetZ());
00576       glVertex3f(sBBox.MaxCorner.GetX(), sBBox.MaxCorner.GetY(), sBBox.MinCorner.GetZ());
00577       glVertex3f(sBBox.MaxCorner.GetX(), sBBox.MaxCorner.GetY(), sBBox.MaxCorner.GetZ());
00578       glVertex3f(sBBox.MaxCorner.GetX(), sBBox.MinCorner.GetY(), sBBox.MaxCorner.GetZ());
00579       /* West face */
00580       glNormal3f(0.0f, 1.0f, 0.0f);
00581       glVertex3f(sBBox.MinCorner.GetX(), sBBox.MaxCorner.GetY(), sBBox.MinCorner.GetZ());
00582       glVertex3f(sBBox.MinCorner.GetX(), sBBox.MaxCorner.GetY(), sBBox.MaxCorner.GetZ());
00583       glVertex3f(sBBox.MaxCorner.GetX(), sBBox.MaxCorner.GetY(), sBBox.MaxCorner.GetZ());
00584       glVertex3f(sBBox.MaxCorner.GetX(), sBBox.MaxCorner.GetY(), sBBox.MinCorner.GetZ());
00585       glEnd();
00586       glEnable(GL_LIGHTING);
00587       glLineWidth(1.0f);
00588       glPolygonMode(GL_FRONT_AND_BACK, GL_FILL);
00589    }
00590 
00591    /****************************************/
00592    /****************************************/
00593 
00594    void CQTOpenGLWidget::PlayExperiment() {
00595       m_bFastForwarding = false;
00596       if(nTimerId != -1) killTimer(nTimerId);
00597       nTimerId = startTimer(CPhysicsEngine::GetSimulationClockTick() * 1000.0f);
00598    }
00599 
00600    /****************************************/
00601    /****************************************/
00602 
00603    void CQTOpenGLWidget::FastForwardExperiment() {
00604       m_nFrameCounter = 0;
00605       m_bFastForwarding = true;
00606       if(nTimerId != -1) killTimer(nTimerId);
00607       nTimerId = startTimer(1);
00608    }
00609 
00610    /****************************************/
00611    /****************************************/
00612 
00613    void CQTOpenGLWidget::PauseExperiment() {
00614       m_bFastForwarding = false;
00615       if(nTimerId != -1) killTimer(nTimerId);
00616       nTimerId = -1;
00617    }
00618 
00619    /****************************************/
00620    /****************************************/
00621 
00622    void CQTOpenGLWidget::StepExperiment() {
00623       if(!m_cSimulator.IsExperimentFinished()) {
00624          m_cSimulator.UpdateSpace();
00625          if(m_bFastForwarding) {
00626             /* Frame dropping happens only in fast-forward */
00627             m_nFrameCounter = m_nFrameCounter % m_nDrawFrameEvery;
00628             if(m_nFrameCounter == 0) {
00629                update();
00630             }
00631             ++m_nFrameCounter;
00632          } else {
00633             update();
00634          }
00635          emit StepDone(m_cSpace.GetSimulationClock());
00636       }
00637       else {
00638          PauseExperiment();
00639          emit ExperimentDone();
00640       }
00641    }
00642 
00643    /****************************************/
00644    /****************************************/
00645 
00646    void CQTOpenGLWidget::ResetExperiment() {
00647       m_cSimulator.Reset();
00648       delete m_pcGroundTexture;
00649       if(m_bUsingFloorTexture) delete m_pcFloorTexture;
00650       initializeGL();
00651       update();
00652    }
00653 
00654    /****************************************/
00655    /****************************************/
00656 
00657    void CQTOpenGLWidget::SetDrawFrameEvery(SInt32 n_every) {
00658       m_nDrawFrameEvery = n_every;
00659    }
00660 
00661    /****************************************/
00662    /****************************************/
00663 
00664    void CQTOpenGLWidget::SetGrabFrame(bool b_grab_on) {
00665       m_sFrameGrabData.Grabbing = b_grab_on;
00666    }
00667 
00668    /****************************************/
00669    /****************************************/
00670 
00671    void CQTOpenGLWidget::SetCamera(int n_camera) {
00672       m_cCamera.SetActiveSettings(n_camera);
00673       update();
00674       QToolTip::showText(pos() + geometry().center(), QString("Current camera: #%1").arg(n_camera+1));
00675    }
00676 
00677    /****************************************/
00678    /****************************************/
00679 
00680    void CQTOpenGLWidget::SetCameraFocalLength(double f_length) {
00681       m_cCamera.GetActiveSettings().LensFocalLength = f_length / 1000.0f;
00682       m_cCamera.GetActiveSettings().CalculateYFieldOfView();
00683       m_cCamera.GetActiveSettings().CalculateSensitivity();
00684       QToolTip::showText(pos() + geometry().center(), QString("Motion sens = %1").arg(m_cCamera.GetActiveSettings().MotionSensitivity));
00685       update();
00686    }
00687 
00688    /****************************************/
00689    /****************************************/
00690 
00691    void CQTOpenGLWidget::KeyPressed(QKeyEvent* pc_event) {
00692       switch(pc_event->key()) {
00693          case Qt::Key_W:
00694          case Qt::Key_Up:
00695             /* Forwards */
00696             m_mapPressedKeys[DIRECTION_FORWARDS] = true;
00697             reactToKeyEvent();
00698             break;
00699          case Qt::Key_S:
00700          case Qt::Key_Down:
00701             /* Backwards */
00702             m_mapPressedKeys[DIRECTION_BACKWARDS] = true;
00703             reactToKeyEvent();
00704             break;
00705          case Qt::Key_A:
00706          case Qt::Key_Left:
00707             /* Left */
00708             m_mapPressedKeys[DIRECTION_LEFT] = true;
00709             reactToKeyEvent();
00710             break;
00711          case Qt::Key_D:
00712          case Qt::Key_Right:
00713             /* Right */
00714             m_mapPressedKeys[DIRECTION_RIGHT] = true;
00715             reactToKeyEvent();
00716             break;
00717          case Qt::Key_E:
00718             /* Up */
00719             m_mapPressedKeys[DIRECTION_UP] = true;
00720             reactToKeyEvent();
00721             break;
00722          case Qt::Key_Q:
00723             /* Up */
00724             m_mapPressedKeys[DIRECTION_DOWN] = true;
00725             reactToKeyEvent();
00726             break;
00727          default:
00728             /* Unknown key */
00729             QOpenGLWidget::keyPressEvent(pc_event);
00730             break;
00731       }
00732    }
00733 
00734    /****************************************/
00735    /****************************************/
00736 
00737    void CQTOpenGLWidget::KeyReleased(QKeyEvent* pc_event) {
00738       switch(pc_event->key()) {
00739          case Qt::Key_W:
00740          case Qt::Key_Up:
00741             /* Forwards */
00742             m_mapPressedKeys[DIRECTION_FORWARDS] = false;
00743             reactToKeyEvent();
00744             break;
00745          case Qt::Key_S:
00746          case Qt::Key_Down:
00747             /* Backwards */
00748             m_mapPressedKeys[DIRECTION_BACKWARDS] = false;
00749             reactToKeyEvent();
00750             break;
00751          case Qt::Key_A:
00752          case Qt::Key_Left:
00753             /* Left */
00754             m_mapPressedKeys[DIRECTION_LEFT] = false;
00755             reactToKeyEvent();
00756             break;
00757          case Qt::Key_D:
00758          case Qt::Key_Right:
00759             /* Right */
00760             m_mapPressedKeys[DIRECTION_RIGHT] = false;
00761             reactToKeyEvent();
00762             break;
00763          case Qt::Key_E:
00764             /* Up */
00765             m_mapPressedKeys[DIRECTION_UP] = false;
00766             reactToKeyEvent();
00767             break;
00768          case Qt::Key_Q:
00769             /* Up */
00770             m_mapPressedKeys[DIRECTION_DOWN] = false;
00771             reactToKeyEvent();
00772             break;
00773          default:
00774             /* Unknown key */
00775             QOpenGLWidget::keyPressEvent(pc_event);
00776             break;
00777       }
00778    }
00779 
00780    /****************************************/
00781    /****************************************/
00782 
00783    void CQTOpenGLWidget::DrawArena() {
00784       CVector3 cArenaSize(m_cSpace.GetArenaSize());
00785       CVector3 cArenaMinCorner(m_cSpace.GetArenaCenter().GetX() - cArenaSize.GetX() * 0.5f,
00786                                m_cSpace.GetArenaCenter().GetY() - cArenaSize.GetY() * 0.5f,
00787                                m_cSpace.GetArenaCenter().GetZ() - cArenaSize.GetZ() * 0.5f);
00788       CVector3 cArenaMaxCorner(m_cSpace.GetArenaCenter().GetX() + cArenaSize.GetX() * 0.5f,
00789                                m_cSpace.GetArenaCenter().GetY() + cArenaSize.GetY() * 0.5f,
00790                                m_cSpace.GetArenaCenter().GetZ() + cArenaSize.GetZ() * 0.5f);
00791       /* Disable lighting - no funny color effects */
00792       glDisable(GL_LIGHTING);
00793       /* Enable textures */
00794       glEnable(GL_TEXTURE_2D);
00795       /* Take care of the floor entity if necessary */
00796       /* The texture covers the object like a decal */
00797       glTexEnvi(GL_TEXTURE_ENV, GL_TEXTURE_ENV_MODE, GL_DECAL);
00798 #ifdef ARGOS_WITH_FREEIMAGE
00799       if(m_bUsingFloorTexture) {
00800          /* Use the image as texture */
00801          if(m_cSpace.GetFloorEntity().HasChanged()) {
00802             /* Create an image to use as texture */
00803             m_pcFloorTexture->destroy();
00804             m_pcFloorTexture->create();
00805             m_cSpace.GetFloorEntity().SaveAsImage("/tmp/argos_floor.png");
00806             m_pcFloorTexture->setData(QImage("/tmp/argos_floor.png"));
00807             m_pcFloorTexture->setMinMagFilters(QOpenGLTexture::LinearMipMapLinear,
00808                                                QOpenGLTexture::Linear);
00809             /* Clear the 'changed' flag on the floor entity */
00810             m_cSpace.GetFloorEntity().ClearChanged();
00811          }
00812          /* Draw the floor entity along with its texture */
00813          m_pcFloorTexture->bind();
00814          glBegin(GL_QUADS);
00815          glTexCoord2d(0.0f, 1.0f); glVertex3f(cArenaMinCorner.GetX(), cArenaMinCorner.GetY(), 0.0f);
00816          glTexCoord2d(1.0f, 1.0f); glVertex3f(cArenaMaxCorner.GetX(), cArenaMinCorner.GetY(), 0.0f);
00817          glTexCoord2d(1.0f, 0.0f); glVertex3f(cArenaMaxCorner.GetX(), cArenaMaxCorner.GetY(), 0.0f);
00818          glTexCoord2d(0.0f, 0.0f); glVertex3f(cArenaMinCorner.GetX(), cArenaMaxCorner.GetY(), 0.0f);
00819          glEnd();
00820       }
00821       else {
00822 #endif
00823          /* Wrap the texture at the edges, which in this case means that it is repeated */
00824          glTexParameteri(GL_TEXTURE_2D, GL_TEXTURE_WRAP_S, GL_REPEAT);
00825          glTexParameteri(GL_TEXTURE_2D, GL_TEXTURE_WRAP_T, GL_REPEAT);
00826          m_pcGroundTexture->bind();
00827          /* Draw the floor along with its texture */
00828          glBegin(GL_QUADS);
00829          glTexCoord2f(0.0f, cArenaSize.GetY());              glVertex3f(cArenaMinCorner.GetX(), cArenaMinCorner.GetY(), 0.0f);
00830          glTexCoord2f(cArenaSize.GetX(), cArenaSize.GetY()); glVertex3f(cArenaMaxCorner.GetX(), cArenaMinCorner.GetY(), 0.0f);
00831          glTexCoord2f(cArenaSize.GetX(), 0.0f);              glVertex3f(cArenaMaxCorner.GetX(), cArenaMaxCorner.GetY(), 0.0f);
00832          glTexCoord2f(0.0f, 0.0f);                           glVertex3f(cArenaMinCorner.GetX(), cArenaMaxCorner.GetY(), 0.0f);
00833          glEnd();
00834 #ifdef ARGOS_WITH_FREEIMAGE
00835       }
00836 #endif
00837       /* Disable the textures */
00838       glDisable(GL_TEXTURE_2D);
00839       /* Draw walls */
00840       glDisable(GL_CULL_FACE);
00841       glPolygonMode(GL_FRONT_AND_BACK, GL_LINE);
00842       glLineWidth(10.0f);
00843       glColor3f(0.0f, 0.0f, 0.0f);
00844       /* This part covers the top and bottom faces (parallel to XY) */
00845       glBegin(GL_QUADS);
00846       /* Top face */
00847       glVertex3f(cArenaMinCorner.GetX(), cArenaMinCorner.GetY(), cArenaMaxCorner.GetZ());
00848       glVertex3f(cArenaMaxCorner.GetX(), cArenaMinCorner.GetY(), cArenaMaxCorner.GetZ());
00849       glVertex3f(cArenaMaxCorner.GetX(), cArenaMaxCorner.GetY(), cArenaMaxCorner.GetZ());
00850       glVertex3f(cArenaMinCorner.GetX(), cArenaMaxCorner.GetY(), cArenaMaxCorner.GetZ());
00851       glEnd();
00852       /* This part covers the faces (South, East, North, West) */
00853       glBegin(GL_QUADS);
00854       /* South face */
00855       glVertex3f(cArenaMinCorner.GetX(), cArenaMinCorner.GetY(), cArenaMinCorner.GetZ());
00856       glVertex3f(cArenaMinCorner.GetX(), cArenaMinCorner.GetY(), cArenaMaxCorner.GetZ());
00857       glVertex3f(cArenaMinCorner.GetX(), cArenaMaxCorner.GetY(), cArenaMaxCorner.GetZ());
00858       glVertex3f(cArenaMinCorner.GetX(), cArenaMaxCorner.GetY(), cArenaMinCorner.GetZ());
00859       /* East face */
00860       glVertex3f(cArenaMinCorner.GetX(), cArenaMinCorner.GetY(), cArenaMinCorner.GetZ());
00861       glVertex3f(cArenaMaxCorner.GetX(), cArenaMinCorner.GetY(), cArenaMinCorner.GetZ());
00862       glVertex3f(cArenaMaxCorner.GetX(), cArenaMinCorner.GetY(), cArenaMaxCorner.GetZ());
00863       glVertex3f(cArenaMinCorner.GetX(), cArenaMinCorner.GetY(), cArenaMaxCorner.GetZ());
00864       /* North face */
00865       glVertex3f(cArenaMaxCorner.GetX(), cArenaMinCorner.GetY(), cArenaMinCorner.GetZ());
00866       glVertex3f(cArenaMaxCorner.GetX(), cArenaMaxCorner.GetY(), cArenaMinCorner.GetZ());
00867       glVertex3f(cArenaMaxCorner.GetX(), cArenaMaxCorner.GetY(), cArenaMaxCorner.GetZ());
00868       glVertex3f(cArenaMaxCorner.GetX(), cArenaMinCorner.GetY(), cArenaMaxCorner.GetZ());
00869       /* West face */
00870       glVertex3f(cArenaMinCorner.GetX(), cArenaMaxCorner.GetY(), cArenaMinCorner.GetZ());
00871       glVertex3f(cArenaMinCorner.GetX(), cArenaMaxCorner.GetY(), cArenaMaxCorner.GetZ());
00872       glVertex3f(cArenaMaxCorner.GetX(), cArenaMaxCorner.GetY(), cArenaMaxCorner.GetZ());
00873       glVertex3f(cArenaMaxCorner.GetX(), cArenaMaxCorner.GetY(), cArenaMinCorner.GetZ());
00874       glEnd();
00875       glLineWidth(1.0f);
00876       glPolygonMode(GL_FRONT_AND_BACK, GL_FILL);
00877       glEnable(GL_CULL_FACE);
00878       /* Restore lighting */
00879       glEnable(GL_LIGHTING);
00880    }
00881 
00882    /****************************************/
00883    /****************************************/
00884 
00885    void CQTOpenGLWidget::DrawAxes() {
00886    }
00887 
00888    /****************************************/
00889    /****************************************/
00890 
00891    void CQTOpenGLWidget::timerEvent(QTimerEvent* pc_event) {
00892       StepExperiment();
00893    }
00894 
00895    /****************************************/
00896    /****************************************/
00897 
00898    void CQTOpenGLWidget::mousePressEvent(QMouseEvent* pc_event) {
00899       /*
00900        * Mouse press without shift
00901        * Either pure press, or press + CTRL
00902        */
00903       if(! (pc_event->modifiers() & Qt::ShiftModifier)) {
00904          m_bMouseGrabbed = true;
00905          m_cMouseGrabPos = pc_event->pos();
00906       }
00907       /*
00908        * Mouse press with shift
00909        */
00910       else {
00911          m_bMouseGrabbed = false;
00912          SelectInScene(pc_event->pos().x(),
00913                        pc_event->pos().y());
00914       }
00915    }
00916 
00917    /****************************************/
00918    /****************************************/
00919 
00920    void CQTOpenGLWidget::mouseReleaseEvent(QMouseEvent* pc_event) {
00921       /*
00922        * Mouse grabbed, selected entity, CTRL pressed
00923        */
00924       if(m_bMouseGrabbed &&
00925          m_sSelectionInfo.IsSelected &&
00926          (pc_event->modifiers() & Qt::ControlModifier)) {
00927          /* Treat selected entity as an embodied entity */
00928          CEmbodiedEntity* pcEntity = dynamic_cast<CEmbodiedEntity*>(
00929             m_cSpace.GetRootEntityVector()[m_sSelectionInfo.Index]);
00930          if(pcEntity == NULL) {
00931             /* Treat selected entity as a composable entity with an embodied component */
00932             CComposableEntity* pcCompEntity = dynamic_cast<CComposableEntity*>(
00933                m_cSpace.GetRootEntityVector()[m_sSelectionInfo.Index]);
00934             if(pcCompEntity != NULL && pcCompEntity->HasComponent("body")) {
00935                pcEntity = &pcCompEntity->GetComponent<CEmbodiedEntity>("body");
00936             }
00937             else {
00938                /* All conversions failed, get out */
00939                m_bMouseGrabbed = false;
00940                return;
00941             }
00942          }
00943          /*
00944           * If we get here, pcEntity is set to a non-NULL value
00945           * Move the entity to the wanted place
00946           */
00947          /* Create a plane coincident with the world XY plane, centered at the entity position */
00948          CPlane cXYPlane(pcEntity->GetOriginAnchor().Position, CVector3::Z);
00949          /* Create a ray from the image pixel to the world */
00950          CRay3 cMouseRay =
00951             RayFromWindowCoord(pc_event->pos().x(),
00952                                pc_event->pos().y());
00953          /* Calculate the new entity position as the intersection of the projected mouse position
00954             with the plane created before */
00955          CVector3 cNewPos;
00956          if(cMouseRay.Intersects(cXYPlane, cNewPos)) {
00957             pcEntity->MoveTo(cNewPos,
00958                              pcEntity->GetOriginAnchor().Orientation);
00959             /* Entity moved, redraw */
00960             update();
00961          }
00962       }
00963       /*
00964        * Mouse was grabbed, button released -> ungrab mouse
00965        */
00966       m_bMouseGrabbed = false;
00967    }
00968 
00969    /****************************************/
00970    /****************************************/
00971 
00972    void CQTOpenGLWidget::mouseMoveEvent(QMouseEvent* pc_event) {
00973       /*
00974        * Moving while mouse grabbed -> camera movement
00975        */
00976       if(m_bMouseGrabbed) {
00977          if(! (pc_event->modifiers() & Qt::ControlModifier)) {
00978             /*
00979              * Camera movement
00980              */
00981             if(pc_event->buttons() == Qt::LeftButton) {
00982                if (m_bInvertMouse) m_cCamera.Rotate( pc_event->pos() - m_cMouseGrabPos);
00983                else m_cCamera.Rotate( m_cMouseGrabPos - pc_event->pos());
00984                m_cMouseGrabPos = pc_event->pos();
00985                update();
00986             }
00987             else if(pc_event->buttons() == Qt::RightButton) {
00988                QPoint cDelta(pc_event->pos() - m_cMouseGrabPos);
00989                m_cCamera.Move(-cDelta.y(), cDelta.x(), 0);
00990                m_cMouseGrabPos = pc_event->pos();
00991                update();
00992             }
00993             else if(pc_event->buttons() == Qt::MidButton) {
00994                QPoint cDelta(pc_event->pos() - m_cMouseGrabPos);
00995                m_cCamera.Move(0, 0, cDelta.y());
00996                m_cMouseGrabPos = pc_event->pos();
00997                update();
00998             }
00999          }
01000       }
01001    }
01002 
01003    /****************************************/
01004    /****************************************/
01005 
01006    void CQTOpenGLWidget::keyPressEvent(QKeyEvent* pc_event) {
01007       m_cUserFunctions.KeyPressed(pc_event);
01008    }
01009 
01010    /****************************************/
01011    /****************************************/
01012 
01013    void CQTOpenGLWidget::keyReleaseEvent(QKeyEvent* pc_event) {
01014       m_cUserFunctions.KeyReleased(pc_event);
01015    }
01016 
01017    /****************************************/
01018    /****************************************/
01019 
01020    void CQTOpenGLWidget::reactToKeyEvent() {
01021       SInt32 nForwardsBackwards = 0;
01022       SInt32 nSideways = 0;
01023       SInt32 nUpDown = 0;
01024 
01025       if(m_mapPressedKeys[DIRECTION_UP])        nUpDown++;
01026       if(m_mapPressedKeys[DIRECTION_DOWN])      nUpDown--;
01027       if(m_mapPressedKeys[DIRECTION_LEFT])      nSideways++;
01028       if(m_mapPressedKeys[DIRECTION_RIGHT])     nSideways--;
01029       if(m_mapPressedKeys[DIRECTION_FORWARDS])  nForwardsBackwards++;
01030       if(m_mapPressedKeys[DIRECTION_BACKWARDS]) nForwardsBackwards--;
01031 
01032       if(nForwardsBackwards != 0 ||
01033          nSideways != 0 ||
01034          nUpDown != 0) {
01035          m_cCamera.Move(15 * nForwardsBackwards,
01036                         15 * nSideways,
01037                         15 * nUpDown);
01038          update();
01039       }
01040    }
01041 
01042    /****************************************/
01043    /****************************************/
01044 
01045    void CQTOpenGLWidget::resizeEvent(QResizeEvent* pc_event) {
01046       /* Call parent's resize event handler */
01047       QOpenGLWidget::resizeEvent(pc_event);
01048       /* Show new window size */
01049       QToolTip::showText(pos() + geometry().center(), QString("Size: %1 x %2").arg(pc_event->size().width()).arg(pc_event->size().height()));
01050    }
01051 
01052    /****************************************/
01053    /****************************************/
01054 
01055    void CQTOpenGLWidget::SFrameGrabData::Init(TConfigurationNode& t_tree) {
01056       if(NodeExists(t_tree, "frame_grabbing")) {
01057          TConfigurationNode& tNode = GetNode(t_tree, "frame_grabbing");
01058          std::string strBuffer;
01059          /* Parse directory, removing trailing '/' */
01060          strBuffer = ".";
01061          GetNodeAttributeOrDefault(tNode, "directory", strBuffer, strBuffer);
01062          size_t unEndPos = strBuffer.find_last_not_of("/ \t");
01063          if(unEndPos != std::string::npos) {
01064             strBuffer = strBuffer.substr(0, unEndPos+1);
01065          }
01066          Directory = strBuffer.c_str();
01067          QDir cDirectory(Directory);
01068          if(!cDirectory.exists()) {
01069             THROW_ARGOSEXCEPTION("QTOpenGL: frame grabbing directory \"" << strBuffer << "\" does not exist. Create it first!");
01070          }
01071          /* Parse base name */
01072          strBuffer = "frame_";
01073          GetNodeAttributeOrDefault(tNode, "base_name", strBuffer, strBuffer);
01074          BaseName = strBuffer.c_str();
01075          /* Parse format */
01076          strBuffer = "png";
01077          GetNodeAttributeOrDefault(tNode, "format", strBuffer, strBuffer);
01078          Format = strBuffer.c_str();
01079          /* Parse quality */
01080          GetNodeAttributeOrDefault(tNode, "quality", Quality, Quality);
01081       }
01082    }
01083 
01084    /****************************************/
01085    /****************************************/
01086 
01087 }
```

---

Generated on 10 Jul 2018 for ARGoS by 
 1.6.1
